# Toxin Analysis of Freshwater Cyanobacterial and Marine Harmful Algal Blooms on the West Coast of Florida and Implications for Estuarine Environments

**DOI:** 10.1007/s12640-020-00248-3

**Published:** 2020-07-18

**Authors:** J. S. Metcalf, S. A. Banack, R. A. Wessel, M. Lester, J. G. Pim, J. R. Cassani, P. A. Cox

**Affiliations:** 1 Brain Chemistry Labs, Jackson, WY 83001 USA; 2 Sanibel-Captiva Conservation Foundation, Sanibel, FL 33957 USA; 3 Path of Wellness Holistic Health, Lexington, GA 30648 USA; 4 Calusa Waterkeeper, Inc., PO Box 1165, Fort Myers, FL 33902 USA

**Keywords:** Red tide, Cyanobacteria, Microcystin, Brevetoxin, Exposure, Monitoring

## Abstract

Recent marine and freshwater algal and cyanobacterial blooms in Florida have increased public concern and awareness of the risks posed by exposure to these organisms. In 2018, Lake Okeechobee and the Caloosahatchee river, on the west coast of Florida, experienced an extended bloom of *Microcystis* spp. and a bloom of *Karenia brevis* in the coastal waters of the Gulf of Mexico that coincided in the Fort Myers area. Samples from the Caloosahatchee at Fort Myers into Pine Island Sound and up to Boca Grande were collected by boat. High concentrations of microcystin-LR were detected in the cyanobacterial bloom along with brevetoxins in the marine samples. Furthermore, β-N-methylamino-L-alanine (BMAA) and isomers *N*-(2-aminoethyl)glycine (AEG) and 2,4-diaminobuytric acid (DAB) were detected in marine diatoms and dinoflagellates, and cyanobacteria of freshwater origin. High freshwater flows pushed the cyanobacterial bloom to barrier island beaches and *Microcystis* and microcystins could be detected into the marine environment at a salinity of 41 mS/cm. For comparison, in 2019 collections of *Dapis* (a new generic segregate from *Lyngbya*) mats from Sarasota showed high concentrations of BMAA, suggesting the possibility of long-term exposure of residents to BMAA. The findings highlight the potential for multiple, potentially toxic blooms to co-exist and the possible implications for human and animal health.

## Introduction

Cyanobacteria are prokaryotes taxonomically placed within a phylum of Gram-negative bacteria that occur in marine, terrestrial, and freshwater environments (Fogg et al. 1973). Under conditions of excess nutrients such as nitrogen and/or phosphorous, they can form mass populations or blooms in aquatic environments (Smith et al. 1999). When such conditions allow, these mass populations can be considerable in size and with the action of calm weather and wind can form scums on shorelines and in embayments (Metcalf et al. 2018). Although esthetically unpleasant, of bigger concern is their potential to produce highly potent, low molecular weight toxins with acute and chronic human health impacts. The toxins produced can range from hepatotoxins, such as microcystins (MC) and nodularins, to cytotoxins including cylindrospermopsins, and neurotoxins such as anatoxin-a and anatoxin-a(s), as well as the more recently characterized β-N-methylamino-L-alanine (BMAA) (Metcalf and Codd 2012). Furthermore, research is increasingly expanding the known toxicological modes of action of cyanobacterial toxins, such that for example, microcystins and cylindrospermopsin are being considered neurotoxins (Hu et al. 2016; Hinojosa et al. 2019; Wang et al. 2019). These new understandings will also have implications concerning future risk assessment of blooms.

Although cyanobacteria are often referred to as blue-green algae, they are true bacteria, and are different from algal groups such as diatoms, dinoflagellates, and haptophytes that are also capable of producing a wide range of toxic compounds in aquatic environments, as evidenced by periodic fish kills (Burkholder 1981; Hallegraeff 2003). The majority of such diatom and dinoflagellate blooms have the potential to contaminate shellfish, resulting in economic losses and fisheries closures (Hoagland and Scatasta 2006). One dinoflagellate of historic concern is *Karenia brevis,* which is capable of producing brevetoxins, a family of complex volatile polyether toxins and sodium channel blocking compounds (Landsberg et al. 2009). In the Gulf of Mexico, periodic blooms of *Karenia brevis* occur as red tides that can kill marine shellfish, fish, and mammals (Magaña et al. 2003). For the duration of these blooms, consumption of marine animals and their flesh can lead to further intoxications and illness (James et al. 2010).

Although marine and freshwater harmful algal or cyanobacterial blooms can occur, they are not often concomitant. When this happens, human and animal health may be at further risk of adverse effects by synergism between multiple classes of aquatic toxins (e.g. Fire et al. 2011; Peacock et al. 2018). Barriers that prevent blooms from co-occurring are often physical and include salinity, which have been shown to control blooms of freshwater cyanobacteria when they enter marine waters (Preece et al. 2017).

Further examination of cyanobacteria and microalgae continues to discover novel compounds with adverse biological activity such as euglenophycin in *Euglena* (Zimba et al. 2010). Research concerning cyanobacteria has identified a novel non-protein amino acid with neurotoxic properties termed β-*N-*methylamino-L-alanine (BMAA; Cox et al. 2003; Karamyan and Speth 2008). Evidence indicates that there is the potential for many cyanobacterial genera and species to produce BMAA (Cox et al. 2005; Metcalf et al. 2008) and its isomers, 2,4-diaminobutyric acid (DAB; Banack et al. 2011) and *N-*(2-aminoethyl)glycine (AEG; Banack et al. 2012). Chronic exposure to BMAA has been identified as a risk factor for the development of neurodegenerative diseases such as amyotrophic lateral sclerosis (ALS) and Alzheimer’s disease, as dietary exposure to BMAA can cause neuropathologies consistent with human neurodegenerative disease (Cox et al. 2016; Davis et al. 2020). Although BMAA was originally discovered in the seeds of cycads (Bell 2009), it was subsequently found to be produced by endosymbiotic cyanobacteria of the genus *Nostoc* present in the cycad coralloid roots (Cox et al. 2003). BMAA production has now been demonstrated in diverse taxa of cyanobacteria (Cox et al., 2005), with marine diatoms and dinoflagellates now considered possible sources (Jiang et al. 2014; Lage et al. 2014; Réveillon et al. 2016). Freshwater diatoms have also been shown to contain BMAA (Violi et al. 2019).

Florida’s warm climate and ready supply of nutrients create highly favorable conditions for the production of harmful cyanobacterial and algal blooms, such as the recent blooms of *Microcystis* spp. that have appeared in Lake Okeechobee (Metcalf et al. 2018). Annually during the wet season, discharges from the Lake are made to the western flowing Caloosahatchee and eastern flowing St. Lucie river to lower lake water levels, reducing the risk of water breaching the dikes such as occurred in 1928, when 2500 people were killed in the aftermath of the Okeechobee Hurricane (Rappaport & Fernandez-Partagas 1995). In 2016, discharges of lake water via the St. Lucie River to Stuart, Florida, supported a large cyanobacterial population that was found to contain high concentrations of microcystins, along with detectable concentrations of anatoxin-a(*s*) and BMAA and its isomers (Metcalf et al. 2018). In 2018, a similar situation resulted, with discharges of cyanobacteria to both the St. Lucie Canal and the Caloosahatchee, resulting in a large bloom of cyanobacteria reaching San Carlos Bay, Pine Island Sound and the Gulf of Mexico. These releases coincided with a bloom of brevetoxin-producing *Karenia brevis* in the Gulf of Mexico. The purpose of this study was to sample and analyze red tide and cyanobacterial blooms for BMAA, brevetoxins, and other cyanotoxins, compared with analyses of extensive *Dapis* mats in the Gulf of Mexico near Sarasota in 2019.

## Materials and Methods

### Sampling

**Fig. 1:**
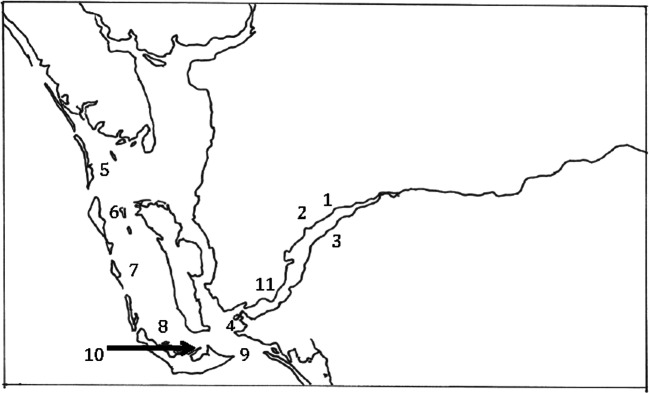
Location of sampling sites in the Caloosahatchee and Pine Island Sound. Numbers correspond to sampling locations in Tables 1, 2, 3

Cyanobacterial and algal samples were collected by boat on 16 August 2018 in Cape Coral, Fort Myers and through Pine Island Sound to Boca Grande (Fig. 1). At each location, GPS coordinates were recorded and a grab sample taken and a 5 min plankton net trawl was carried out. Samples were kept cold and returned to the laboratory for processing and analysis. Samples of *Dapis* (a new generic segment from *Lyngbya;* Engene et al. 2018) mats were collected in August 2019 in Sarasota and transported cold to the laboratory for processing and analysis. At the laboratory, all samples underwent microscopy to determine whether diatoms, dinoflagellates, or cyanobacteria were present, the latter identified to genus/species level (Whitton 2002).

### Extraction of Cyanotoxins

At the laboratory, cyanobacterial material was freeze-dried. Aliquots were removed, weighed, and extracted with 70% methanol (microcystins), DQ water (Anatoxin-a, cylindrospermopsin, anatoxin-a(*S*)), and 20% (v/v) trichloroacetic acid for free BMAA, each with ultrasonication at approximately 50 mg/mL dry weight. Samples were left for 1 h at room temperature (70% methanol, DQ water) or overnight at 4 °C for 20% (v/v) TCA (free BMAA and isomers). The remaining material after TCA extraction underwent digestion in 6 M HCl for 16 h at 110 °C for bound BMAA and isomers as described in Metcalf et al. (2018). After extraction, supernatants of methanolic extractions were dried in a SpeedVac and 20% TCA and 6 M HCl extracts were centrifuge filtered and the filtrate dried in a SpeedVac. Dried and aqueous extracts were stored at − 20 °C until analyzed. For the analysis of brevetoxins by ELISA (Abraxis, Warminster, PA, USA), grab samples were stabilized according to the manufacturer’s instructions.

### Analysis of Microcystins

Microcystins, anatoxin-a, and cylindrospermopsin were analyzed by UPLC-PDA using spectral matching with reference to standards (Metcalf et al. 2018). Dried 70% (v/v) methanol extracts were resuspended in a minimal volume of 70% (v/v) methanol and analyzed by ultra-high performance liquid chromatography with photodiode array detection (UPLC-PDA, Waters Acquity Sample Manager and Binary Solvent Manager, Waters, Milford, MA, USA) following separation with a Waters Acquity Ultra 2.1 × 100 mm C18 column heated to 55 °C. Solvents of purified water + 0.1% (v/v) trifluoroacetic acid (TFA, eluent A) and acetonitrile + 0.1% (v/v) TFA (eluent B) were used for UPLC-PDA analysis, whereas purified water + 0.1% (v/v) formic acid (eluent A) and acetonitrile + 0.1% (v/v) formic acid (eluent B) were used for single quadrupole mass spectrometric analysis. Separation was achieved using a gradient of 25–75% eluent B over 5 min, and monitoring at 238 nm for PDA detection or *m/z* 995.56 using a Waters EMD single quadrupole mass spectrometer to confirm the presence of microcystin-LR after UPLC-PDA analysis. Parameters in the EMD were set at 2.9 kV capillary voltage and 27 V cone voltage, with extractor and Rf lens set at 2 and 0.7 V, respectively. The source temperature was maintained at 130 °C with a desolvation temperature of 400 °C. Nitrogen desolvation gas was supplied at 500 L/h with analyzer settings of 14.4, 15.0, 0.3, and 650 for low-mass resolution, high-mass resolution, ion energy, and multiplier, respectively. UPLC-PDA spectra were compared with a microcystin-LR standard (475815, Millipore-Sigma, St. Louis MO), confirmed by UPLC-EMD, and quantified as microcystin-LR.

### Analysis of Anatoxin-a, Cylindrospermopsin, Anatoxin-a(S), and Brevetoxins

Aqueous extracts of cyanobacteria were analyzed by Waters Acquity UPLC-PDA using a Waters Acquity Ultra 2.1 × 100 mm column maintained at 40 °C using DirectQ (DQ) water + 0.1% TFA (eluent A) and acetonitrile + 0.1% TFA (eluent B) from 0 to 0.5% eluent B over 5 min for anatoxin-a and cylindrospermopsin. Chromatograms were monitored at 227 nm and 262 nm for anatoxin-a and cylindrospermopsin, respectively, and compared with standards of these two cyanotoxins (Metcalf et al. 2018).

Anatoxin-a(*s*) was analyzed in aqueous and methanolic extracts using a colorimetric acetylcholine esterase inhibition assay with reference to a neostigmine standard (Metcalf et al. 2018). Stock solutions of acetylcholinesterase (AChE) (C288, electric eel, type V-S, Sigma, St. Louis, MO, USA) and acetylthiocholine (0.1M) were prepared in 0.1 M KH_2_PO_4_ buffer (pH 8.0) and kept frozen. A 0.01 M solution of 5,5′-Dithiobis(2-nitrobenzoic acid) (D8130, DTNB, Millipore-Sigma, St. Louis, MO) was also prepared in 0.1 M KH_2_PO_4_ buffer, and 15 mg of NaHCO_3_ was added to the final 10 ml volume and kept frozen. Into the wells of a microtiter plate, 300 μL of 0.1 M KH_2_PO_4_ buffer, 20 μL of DTNB, 0.3 μL of sample (or neostigmine standard), 2 μL ATCh, and 10 μL AChE (0.25U) were added in order. The plate was read for 10 min with absorbance measurements every minute at 412 nm using a PowerWave HT Microplate Spectrophotometer (Biotek, Winooski, VT, USA).

Brevetoxins were measured with dilutions of samples using a commercially available ELISA according to the manufacturer’s instructions (520,026, Abraxis, Warminster, PA, USA).

### Analysis of BMAA and Isomers

Free and protein-bound BMAA was analyzed using a Thermo Scientific TSQ Quantiva (Thermo-Fisher Scientific, Waltham, MA, USA) triple quadrupole mass spectrometer with a Thermo Vanquish pump, autosampler, and heating compartment using a fully validated method (Banack 2020). Separation was achieved using a hypersil gold C18 UPLC column (100 × 2.1 mm i.d., 1.9 μm particle size) with 20 mM ammonium acetate (pH 5.0, eluent A) and 100% methanol (eluent B) at a flow rate of 0.5 ml/min and a gradient of 90% eluent A for 1 min, 60% A at 4.8 min, curve 5, 10% A from 5.6 min until 6.8 min before returning to starting conditions at 8 min. Nitrogen was supplied to the mass spectrometer by a Genius 3022 nitrogen generator (Peak Scientific, Billerica, MA). Samples were derivatized with 6-aminoquinolyl-N-hydroxysuccinimidyl carbamate (AQC; Metcalf et al. 2018; Banack, 2020) and supplied to the mass spectrometer using a H-ESI probe in positive mode (3500 V). Spray voltage was static, aux gas (10 arb), sweep gas (0.1 arb), and sheath gas (50 arb) were set, the ion transfer tube (350 °C) and vaporization (400 °C) temperatures were programmed, and resolutions of Q1 and Q3 were both 0.7 FWHM, with a cycle time of 0.4 s and argon CID gas of 2mTorr. A resulting LOD of 0.01 ng/mL and LLOQ of 0.04 ng/mL were calculated following FDA compliance guidelines (FDA 2018).

### Monitoring Data

Salinity measurements were obtained from online in situ meters placed within the Caloosahatchee and Pine Island Sound to provide context to the results of the environmental analyses. Salinity data were recorded hourly at 7 sites in the Caloosahatchee, Pine Island Sound, and Gulf of Mexico. WetLabs WQM sensors were controlled by a Store-X data logger capture and data were sent to a dedicated server (http://recon.sccf.org). The sensor was cleaned monthly and calibrated at WetLabs annually. Anti-fouling measures included the coating of copper-based paints and a tributyltin donut in the intake tubing (Orrico et al. 2007). Salinity values were recorded as parts per thousand (ppt) and converted to mS/cm.

## Results

**Fig. 2:**
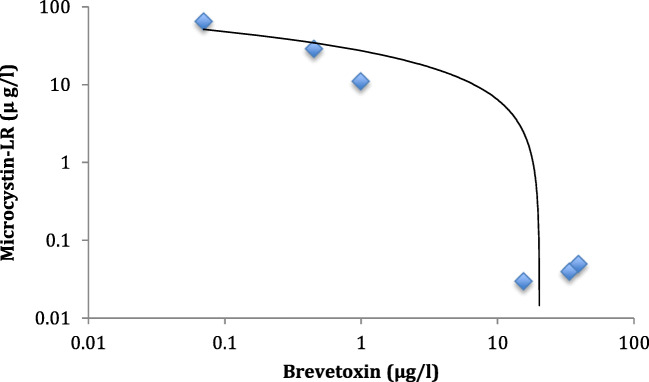
Regression analysis of brevetoxin and microcystin concentrations when co-occurring in samples obtained from the Caloosahatchee and Pine Island Sound. For values, see Table 1 and the regression line is shown (y = − 9.405log(x) + 27.231; *R*^2^ = 0.84)

**Fig. 3:**
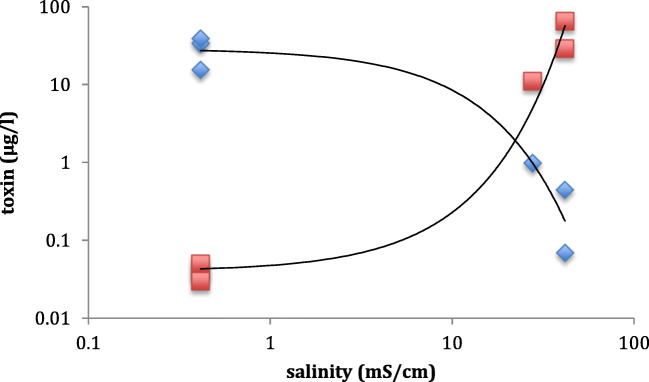
Regression analysis of salinity and brevetoxin and microcystin-LR concentrations in samples from the Caloosahatchee and Pine Island Sound. The cross-over point corresponds to the tideline at Punta Rassa. Regression lines are shown for microcystin-LR (diamond; y = 28.752e^-0.122x^, *R*^2^ = 0.94) and brevetoxin (square; y = 0.0399e^0.1741x^, *R*^2^ = 0.98)

Analysis of the cyanobacterial bloom showed the presence of microcystins by UPLC-PDA, verified as MC-LR using UPLC-MS (Table 1) with a good correlation between MC values determined using PDA and MS (R^2^ = 0.99). Microcystins were found to co-occur with brevetoxins in some samples and a logarithmic relationship was observed between mean values (Table 1, Fig. 2). Microcystins were also present in areas of low and intermediate salinity, and inverse relationships were observed between brevetoxins and microcystins with respect to salinity, with decreasing concentrations of MC-LR with increasing salinity (up to 41 mS/cm), and decreasing brevetoxins with decreasing salinity, although very low concentrations of brevetoxins could be detected by ELISA at salinities of 0.4 mS/cm within the Fort Myers area (Fig. 3).

**Table 1:** Analysis of microcystins, anatoxin-a(S), and brevetoxins in comparison with salinity values obtained from in situ probes. Values are expressed as μg/L of water and values in parentheses are expressed as μg/mg

Site	Microscopy	MC (PDA)	No. Var.	MC (MS)	%LR	ATXa(s)	Brevetox	Salinity mS/cm
1	M	3707.42 (3.78)	1	2833 (2.89)	100	ND	0.21	0.41
2	M,A,D,Di	63.46 (4.60)	1	39.10 (3.17)	100	ND	0.05	0.41
3	M,D,GA	14.55 (0.81)	1	15.48 (0.86)	100	ND	0.03	0.41
4	M,D,Di	0.89 (0.02)	1	0.99 (0.02)	100	ND	11.09	27.67
5	D,Di	ND	-	ND	-	2	0.41	NA
6	D,C	ND	-	ND	-	ND	28.96	NA
7	C,D,Di	ND	-	ND	-	ND	11.29	NA
8	M,D,Di	0.34 (0.002)	1	0.45 (0.003)	100	ND	28.96	NA
9	M,D,Di	ND	-	0.07 (0.0005)	100	ND	64.84	41.67
10	M,D,Di	NP	-	NP	-	2	226.2	41.73

*M*, *Microcystis*; *A*, *Dolichospermum*; *D*, diatoms; *Di*, dinoflagellates; *GA*, green algae; *C*, unidentified cyanobacteria; *NP*, not performed due to insufficient material.; *ND*, not detected; *NA*, not available; *Brevetox*, brevetoxin concentration determined by ELISA. Salinity data is based upon measurements from buoys

BMAA and isomers were detected at all sampling locations, within marine and freshwater environments. The concentrations found as a function of water volume were low, at ng/L concentrations (Table 2), but detectable by triple quadrupole mass spectrometry after derivatization with AQC. Of the isomers detected, DAB was found at higher concentration than AEG, which was also recorded at higher concentration than BMAA. As a function of dry weight, BMAA and isomers were detectable, from ND to 158 ng/g for BMAA, ND to 273 ng/g for AEG, and ND to 3137 ng/g for DAB (Table 3). At sites where no cyanobacteria were recorded (sites 5–7), BMAA and isomers were detected in marine material which comprised diatoms and dinoflagellates according to light microscopy, with bound BMAA concentrations as high as 61 ng/g detected (Table 3). Analyses of *Dapis* mats from Sarasota recorded the presence of high concentrations of BMAA by triple quadrupole mass spectrometry with concentrations as high as 14 and 124 μg/g as a free and protein bound compound, respectively. In this mat material, AEG and DAB were also detected at high concentrations, co-occurring with BMAA.

**Table 2:** Volumetric analysis of water samples for β-*N*-methylamino-L-alanine (BMAA), *N*-(2-aminoethyl)glycine (AEG), and 2,4-diaminobuytric acid (DAB) as free and bound compounds in samples collected from the Caloosahatchee and Pine Island Sound. Values are expressed as ng/L

Location	Free DAB	Bound DAB	Free AEG	Bound AEG	Free BMAA	Bound BMAA
1	106.1	459.9	21.7	11.6	ND	ND
2	1.4	3.3	0.2	0.1	NQ	ND
3	2.0	102.2	4.9	1.1	NQ	2.8
4	1.4	7.0	ND	0.3	NQ	0.2
5	2.4	51.9	ND	0.3	ND	1.0
6	13.7	215.6	1.2	0.9	NQ	5.2
7	6.3	340.9	ND	2.1	0.5	9.3
8	12.9	296.6	1.4	1.6	NQ	5.8
9	13.8	93.4	ND	1.0	NQ	2.6

*ND*, not detected; *NQ*, above the limit of detection but below lower limit of quantification

**Table 3:** Gravimetric analysis of marine and freshwater material for β-*N*-methylamino-L-alanine (BMAA), *N*-(2-aminoethyl)glycine (AEG), and 2,4-diaminobuytric acid (DAB). Values are expressed as ng/g with the exception of the *Dapis* mat material which are expressed as μg/g

Location	Free DAB	Bound DAB	Free AEG	Bound AEG	Free BMAA	Bound BMAA
1	108	469	22	12	ND	ND
2	135	313	22	12	NQ	ND
3	110	6	273	59	NQ	158
4	31	153	ND	7	NQ	3
5	23	500	ND	3	ND	10
6	98	1540	9	6	NQ	37
7	42	2272	ND	14	3	61
8	90	2076	10	11	NQ	40
9	98	659	ND	7	NQ	18
10	41	3137	ND	17	ND	73
11	ND	16	6	25	58	122
*Dapis* mat Sarasota 2019						
Mat 1	ND	8742	86	ND	14	124
Mat 2	59	3112	29	ND	NQ	67
Mat 3	60	2865	11	ND	NQ	36

*ND*, not detected; *NP*, not performed; *NQ*, above the limit of detection but below lower limit of quantification. Sarasota mat material was collected by Michelle Lester and Candy Luther

## Discussion

In 2018, large cyanobacterial blooms were observed in Lake Okeechobee, consistent with other years such as 2016 (Metcalf et al. 2018). The design of the south Florida flood control system uses the Caloosahatchee and St. Lucie rivers as discharge outlets to control water levels within Lake Okeechobee. Water is released, predominantly to the west via the Caloosahatchee and to the east down the St. Lucie Canal (Metcalf et al. 2018). In 2018, releases of cyanobacterial-laden freshwater to both rivers transported a large bloom of *Microcystis* spp. Analysis of water from the Caloosahatchee showed high concentrations of microcystin-LR, sufficient to result in adverse human and animal health effects if ingested, based on the known LD_50_ values for this cyanotoxin (Fawell et al. 1999).

At the same time, a bloom of *Karenia brevis* occurred in the Gulf of Mexico, resulting in numerous press reports concerning the mass deaths of marine life. Consequently, at the tideline, the point where the freshwater bloom and brackish waters from the Gulf converge, there was the possibility that both red tide and cyanobacterial organisms could co-exist (location 4, Fig. 1). However, due to the nature of the environments that these organisms exist in, the salinity of the water has the potential to control both populations and largely keep them separate (Preece et al. 2017; Peacock et al. 2018).

As cyanobacteria include freshwater and marine forms, they are potentially susceptible to the osmotic pressures of a saline environment and may end up undergoing plasmolysis in brackish or marine systems (e.g. Mazur-Marzec et al. 2005). Analysis of microcystins and brevetoxins showed that these two toxins were found in both environments, with microcystin-LR present at salinities of up to 41 mS/cm and positive brevetoxin analyses in water at salinities as low as 0.4 mS/cm, although the very low latter brevetoxin value may be a false positive and requires further investigation. However, these data indicate that there is a potential for co-exposure to marine and freshwater toxins in environments close to the tideline. When such conditions occur, then the potential for synergistic toxic effects would be expected which could exacerbate any animal intoxications. Although little research has been carried out concerning this possibility, co-occurrence of cyanobacterial toxins is known (Metcalf et al. 2008), and in the case of, e.g. cyanobacterial LPS and microcystin-LR, pre- or co-exposure to the LPS increases the tolerance of *Artemia salina* and *Daphnia galeata* from the toxic effects of microcystin-LR (Codd et al. 2005). As there is the potential for exposure to a wide variety of toxicants, future responses to bloom events should consider this possibility. Furthermore, chronic cyanotoxins such as BMAA can biomagnify, with the increased concentrations in shellfish threatening human health long after the original cyanobacterial blooms have subsided (Cox et al. 2003; Field et al. 2013; Masseret et al. 2013; Banack et al. 2014).

Although it is unlikely, due to osmotic stresses, that these organisms were actively blooming in their particular adverse environment, the organisms persisted for some distance. The effect of salinity on organisms such as *Microcystis* has been known for some time (Preece et al. 2017). *Microcystis* appears to be one of the more halotolerant cyanobacteria with reports of survival and growth at salinities of 25 parts per thousand (ppt; 39 mS/cm) (Robson and Hamilton 2003), although microcystin excretion is increased and microcystin production declines with salinities greater than 10 ppt (17 mS/cm; Tonk et al. 2007). Furthermore, although not observed in Florida, blooms of *Nodularia spumigena* can occur in brackish waters with the production of nodularin also possible, such as in the Baltic Sea with growth at an optimal salinity of around 11 ppt (19 mS/cm; Möke et al. 2013) or even in hypersaline environments such as the Great Salt Lake in the USA (Roney et al. 2009).

The distribution of cyanobacterial blooms and microcystins 75 miles downstream from Lake Okeechobee to the tideline was influenced by very high seasonal freshwater discharges from the rivers extensive 865,488-acre watershed combined with releases from Lake Okeechobee. Harmful high-volume inflows to the estuary were reported by the USACE on the order of 1 billion gallons per day in 2018 and as high as 17 billion gallons per day in 2017 following Hurricane Irma (J. Cassani, pers. comm.). Therefore, such a large release may extend the cyanobacterial distribution, as although the time normally taken for physical controls such as salinity to act upon the cells is the same, with the higher flow rates, such toxic cyanobacterial populations may increase their geographical distribution.

Cyanobacterial toxins and marine toxins are known to occur, with multiple toxin classes observed in San Francisco Bay, California (Peacock et al. 2018). Analysis of grab samples and of marine bivalves identified the co-occurrence of microcystins and domoic acid at low, but detectable concentrations (Peacock et al. 2018) with four classes of marine and freshwater toxins detected, highlighting the importance of understanding the toxicity implications of multiple aquatic toxin classes and their potential synergistic toxicity effects.

Although some cyanobacterial toxins such as nodularin are known to occur in brackish environments and neurotoxic factors have been reported in marine environments (Hawser et al. 1991; Hawser and Codd 1992), the finding of BMAA within the saline environments reported here may be a function of their occurrence within the *Microcystis* bloom. Therefore, its production by cyanobacteria may be affected by salinity, although further research is required to determine whether this occurs. BMAA and isomers were also detected in brackish waters containing diatoms and dinoflagellates with no observable cyanobacteria. Investigations into marine blooms containing diatoms and dinoflagellates has detected BMAA (Lage et al. 2014; Jiang et al. 2014), although further work is required to understand the occurrence of this cyanobacterial toxin in these algal groups. Assessment of freshwater diatoms has shown BMAA presence in axenic diatom cultures with 4/5 cultures showing the presence of BMAA and AEG and all five having 2,4-DAB (Violi et al. 2019). The fact that BMAA and isomers were detected at low concentrations in suspended pelagic blooms of *Microcystis* suggest that there may be a risk of long-term exposure, as evidenced by the association between exposure to microcystins and primary liver cancer (Ueno et al. 1996; Svircev et al. 2013), and of exposure to BMAA and the occurrence of human neurodegenerative diseases including Alzheimer’s disease and ALS (Cox et al. 2016; Davis et al. 2020). Although the concentrations of BMAA detected from samplings in 2018 were low, collections made in 2019 of *Dapis* mats, which include species formerly placed within the genus *Lyngbya,* showed much higher concentrations of BMAA and isomers. Consequently, monitoring of such mats should be carried out as these are substantial growths, with people residing in their proximity reporting frequent adverse health effects from possible inhalation of factors and/or organisms connected to these mats (M. Lester, pers. comm.). Multiple exposure routes may exist and inhalation of brevetoxins is certainly considered a health hazard, and microcystin inhalation may result in an enhanced toxic effect (e.g. Cheng et al. 2005; Wood and Dietrich 2011). Assessment of cyanobacteria continues to identify the presence of neurotoxic amino acids such as BMAA in cultures and environmental materials (e.g. Spacil et al. 2010; Metcalf et al. 2018), and with increased methodologies such as triple quadrupole mass spectrometry, further insights into factors affecting toxin production may be revealed, such as has been noted with the relationship between nitrogen supply and BMAA concentration (Downing et al. 2011).

The analyses reported here suggest that under certain conditions, such as high water flows, cyanobacterial blooms may extend some distance into marine environments containing detectable cyanotoxins of known health concern. Furthermore, these data suggest that marine blooms of diatoms and dinoflagellates may be capable of producing BMAA and isomers, in addition to marine toxins such as brevetoxin. With the potential for multiple co-occurrence and co-exposure to brevetoxins, microcystins, and BMAA, risk assessment and monitoring, including toxicity assessment of binary and complex mixtures of marine and freshwater toxins, should be performed to understand the risks of exposure and their effects on human and animal health.
